# Dynamic Compression of the Spinal Cord by Paraspinal Muscles following Cervical Laminectomy: Diagnosis Using Flexion-Extension MRI

**DOI:** 10.1155/2015/275623

**Published:** 2015-04-23

**Authors:** Linton T. Evans, S. Scott Lollis

**Affiliations:** Section of Neurosurgery, Dartmouth-Hitchcock Medical Center, Lebanon, NH 03756, USA

## Abstract

*Introduction*. Flexion-extension, or kinematic, MRI has been used to identify dynamic spondylotic spinal cord compression not seen with traditional static MRI. The use of kinematic MRI to diagnose postoperative complications, specifically dynamic compression, is not as well documented. The authors describe a case of dynamic spinal cord compression by the paraspinal muscles causing worsening myelopathy following cervical laminectomy. This was only diagnosed with flexion-extension MRI. *Methods*. The patient was a 90-year-old male presenting to the neurosurgery clinic with functional decline and cervical spondylotic myelopathy. *Results*. A multilevel laminectomy was performed. Following surgery the patient had progressive weakness and worsening myelopathy. No active cord compression was seen on multiple MRIs obtained in a neutral position, and flexion-extension X-rays did not show instability. A kinematic MRI demonstrated dynamic compression of the spinal cord only during neck extension, by the paraspinal muscles. To relieve the compression, the patient underwent an instrumented fusion, with cross-links used to buttress the paraspinal muscles away from the cord. This resulted in neurologic improvement. *Conclusions*. We describe a novel case of spinal cord compression by paraspinal muscles following cervical laminectomy. In individuals with persistent myelopathy or delayed neurologic decline following posterior decompression, flexion-extension MRI may prove useful in diagnosing this potential complication.

## 1. Introduction

Cervical spondylotic myelopathy (CSM) develops through narrowing of the spinal canal and repetitive injury to the spinal cord as a result of osteophyte formation, disc herniation, and ligament hypertrophy. Previous studies using flexion-extension, or kinematic, MRI have demonstrated dynamic compression of the cervical cord and roots with movement of the neck from neutral position [1, 2].

The use of flexion-extension MRI to detect postoperative complications is not as well-described. We report a case of a 90-year-old male with progressively worsened myelopathy following cervical laminectomy for CSM. Postoperative MRI in neutral alignment demonstrated adequate spinal decompression, and flexion-extension X-rays showed no cervical instability. It was only with kinematic MRI that significant compression of the cervical cord by the paraspinal musculature was found during neck extension.

## 2. Case Report

**Figure 1:**
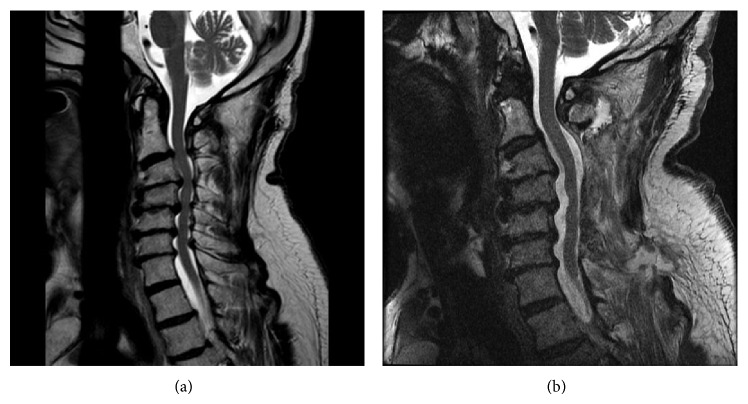
Pre- and postoperative T2-weighted sagittal MRI (static). Postoperative imaging in the neutral position appears to show satisfactory decompression.

A 90-year-old male presented in clinic with functional decline related to worsening right arm weakness, decreased dexterity, and gait instability. He was unable to ambulate without assistance and had difficulty holding objects. Neurologic examination revealed subtle right deltoid weakness and 3/5 strength of the right biceps and brachioradialis. Motor strength was otherwise normal. He walked with a slow and broad-based gait. His reflexes were diffusely absent secondary to generalized peripheral neuropathy. MRI of the cervical spine (Figure 1(a)) revealed severe multilevel stenosis of the spinal canal from C2-3 through C6-7, with mild reversal of the lordotic curvature. Disc degeneration, prominent posterior osteophytes, and hypertrophied ligaments led to CSF effacement and cord compression. No abnormal cord signal was seen on the T2-weighted sequences. There was severe foraminal stenosis at C4-5 and C5-6. On flexion-extension radiographs, the cervical spine was rigid with minimal motion below C2-C3.

Due to the progressive symptoms and functional impairment, surgical intervention was recommended. The patient had significant medical comorbidities, including severe aortic stenosis, hypertension, atherosclerotic coronary artery disease, and chronic obstructive pulmonary disease. Although a ventral approach is typically favored when patients have kyphosis and ventral compression, we did not think that this patient would tolerate a lengthy ventral surgery. A simple laminectomy without fusion was selected because it allowed complete decompression of the stenotic cervical segments, while minimizing operative time; the treating anesthesiologists felt that keeping surgery short would be important in reducing the significant cardiopulmonary risk of general anesthesia. Though the patient did have mild upper cervical kyphosis, we felt that the rigidity of the mid- and lower-cervical spine would prevent significant new deformity and that neither laminoplasty nor instrumented fusion was necessary. The patient therefore underwent posterior decompression of C2-3 through C6-7, with right-sided foraminotomies at C4-5 and C5-6. No intraoperative complications were noted.

Following surgery, the patient developed increased right deltoid weakness (3/5). A postoperative MRI was obtained, demonstrating decompression of the central canal and neural foramina and no evidence of hematoma or other compressive lesion (Figure 1(b)). The weakness was attributed to a C5 syndrome, and he was discharged on postoperative day four. In the ensuing weeks, he experienced progressive weakness of the left arm as well as both lower extremities. His intrinsic hand strength deteriorated to 2/5 on the right and 4/5 on the left, with weakness also noted in hip flexion, hip extension, and knee extension. A Hoffmann sign was now present bilaterally. He described paresthesia throughout the left forearm and hand and diminished sensation in the trunk. He developed new urinary symptoms. He was unable to ambulate.

MRI and flexion-extension radiographs were repeated. There was no evidence of cord compression or cervical instability to account for his decline. On flexion-extension radiographs, minimal motion was noted through the mid- and lower-cervical spine. Formal neurology consultation was obtained. Nerve conduction studies and EMG did not reveal a cause.

**Figure 2:**
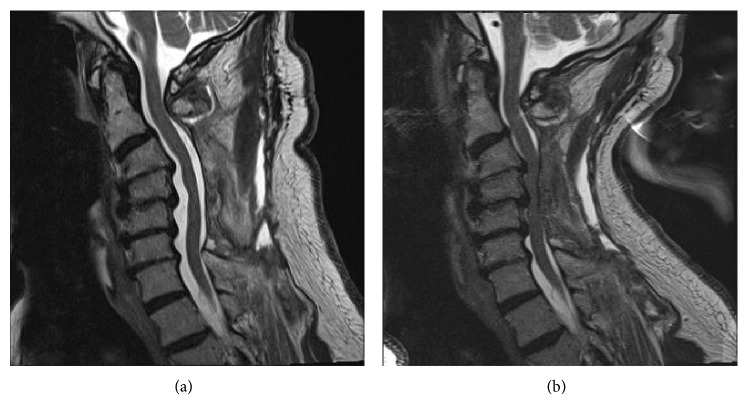
Postoperative T2-weighted sagittal MRI (flexion and extension): spinal cord is compressed by dorsal musculature when the neck is extended. Subaxial spinal alignment (Cobb angle) does not change with flexion-extension, reflecting rigidity of the patient's spondylotic spine.

A flexion-extension MRI of the cervical spine was then performed. The cord was well decompressed in flexion (Figure 2(a)). With neck extension, however, there was significant compression of the cord by the paraspinal musculature at the site of laminectomy (Figure 2(b)). Cobb angle measured from the inferior endplate of C2 to the superior endplate of C7 was 8 degrees for both the flexion and extension images, reflecting the rigidity of the subaxial spine in this patient. Thus, compression did not result from spinal motion* per se*, but rather from anterior translation of the dorsal soft tissue mass into the spinal canal.

To limit further cord compression, the patient underwent a second operation with instrumentation of the lateral masses from C3 through C6 and placement of multiple cross-links. The strategy behind this surgery was not immobilization of the cervical spine through spinal fixation; imaging had already demonstrated the subaxial spine to be completely rigid as a result of spondylosis. The purpose of this surgery was simply to enable cross-link placement. Because cross-links create a rigid barrier between the thecal sac and the overlying musculature, we felt their placement would prevent further repetitive muscular compression. This second procedure was well-tolerated. Routine postoperative imaging demonstrated satisfactory instrumentation. He was discharged to a rehabilitation facility on postoperative day four for continued therapy. At four-week follow-up, he reported moderate improvement. He was able to ambulate seventy feet with a walker and had regained functional use of the left hand and arm. Right arm strength remained significantly impaired. Unfortunately, he died at six weeks following the second operation from an unknown cause. We had hoped to obtain another flexion-extension MRI at three months postoperatively to demonstrate the success of the second surgery in preventing further extension-induced cord compression; however, the patient's untimely death precluded this radiographic documentation.

## 3. Discussion

To our knowledge, this is the first report of dynamic cord compression by paraspinal muscles following cervical laminectomy. Compression occurred with neck extension and was sufficient to produce worsening myelopathy. Spondylosis had rendered the mid- and lower-cervical spine rigid, so that compression resulted from anterior displacement of the musculature against a relatively immobile spine; this is a different pathophysiology than what is more commonly described: a flexion-induced draping of the spinal cord over ventrally positioned disc-osteophyte complexes.

Initially, we considered the possibilities of progressive kyphosis, instability, and ongoing ventral cord compression as the cause of this patient's deterioration. These are far more common causes of clinical worsening after noninstrumented laminectomy, and they occur because the posterior tension band has been destroyed by the surgery. However, the imaging does not support these theories in this case. This patient underwent multiple postoperative flexion-extension X-rays, none of which showed increase in the kyphotic angle from before surgery and none of which showed new listhesis or instability. Indeed, the alignment of the cervical spine remained* identical*, compared to preoperative imaging. This type of spinal rigidity is known to occur in advanced cases of spondylosis with exuberant osteophyte formation, and, in these cases, it can guard against progressive kyphosis.

The unusual phenomenon of dorsal compression by paraspinous musculature was likely enabled by a number of anatomic factors. These include reversal of the lordotic curvature, large disc-osteophyte complexes, a robust posterior cervical muscle mass, and a rigid mid-cervical spine in conjunction with mobile upper segments. The prevalence of this problem after cervical laminectomy is unknown, but it is possible that this pathophysiology accounts for some cases in which patients fail to improve after laminectomy.

Interestingly, this patient did not experience acute symptoms when in a neck-extended posture. Instead, he declined gradually and progressively over a period of weeks. The authors believe that this lack of Lhermitte phenomenon or other acute posture-induced symptoms is not surprising. Typically posture-induced shock-like sensations are caused by intrinsic spinal cord pathology, such as radiation induced myelopathy, transverse myelitis, and chemotherapy-induced myelopathy. While Lhermitte phenomenon has been described in cases of extrinsic compression, as in spondylosis or Chiari malformation; this type of presentation for these entities is considerably rarer. In the authors' experience, extrinsic compression, even when posture-induced, more often results in the gradual, progressive picture noted in this case. While neck flexion might have temporarily relieved acute compression, it would have had no effect on the chronic, repetitive injury that likely was occurring innumerable times each day. Therefore we are not surprised that this patient's postoperative symptoms were not immediately affected by neck posture.

The debate surrounding the optimal surgical strategy for cervical spondylotic myelopathy is ongoing in the medical literature. Anterior decompression is associated with improvement in myelopathy, but it is most commonly used for one- or two-level procedures. Increased rates of pseudarthrosis, adjacent level instability, instrumentation failure, and dysphagia are reported with multilevel anterior procedures [3]. Therefore, posterior decompression is often preferred for multilevel stenosis, particularly when lordosis is maintained.

Cervical laminectomy has been a reliable and longstanding technique for decompression of myelopathic individuals. Perceived disadvantages of this approach include acquired kyphosis, segmental instability, and delayed neurological decline. These concerns have fueled interest in laminoplasty and laminectomy with instrumented fusion. With the exception of a small randomized controlled trial [4], comparisons between the three techniques have been nonrandomized and retrospective, limiting generalizability and leading to continued debate. In a recent survey of thirty academic surgeons, 70% selected cervical laminectomy with instrumented fusion to treat myelopathy associated with multilevel stenosis. Laminoplasty was selected by 23% and laminectomy without fusion by 7% [4]. Thus, the optimal surgical management for cervical myelopathy remains controversial.

We describe a potential complication of cervical laminectomy that may account for some treatment failures. From this single case report, we cannot judge the frequency of this complication, and therefore we cannot offer specific guidance about its role in preoperative decision-making. However, surgeons performing simple cervical laminectomy should be alert to this possibility, so that appropriate imaging with flexion-extension MRI is pursued in a timely fashion.

## 4. Conclusion

Dynamic compression of the spinal cord by paraspinal musculature and dorsal soft tissue is a potential cause of poor outcome following simple cervical laminectomy. Diagnosis requires consideration of dynamic compression and evaluation using flexion-extension MRI.
